# In Vitro Pharmacology of Mitragynine at α‑Adrenoceptors

**DOI:** 10.1021/acschemneuro.5c00719

**Published:** 2025-11-21

**Authors:** Yiming Chen, Jordan Seto, Samuel Obeng, Marco Mottinelli, Sushobhan Mukhopadhyay, Richa Tyagi, Aidan J. Hampson, Christopher R. McCurdy, Lance R. McMahon, Nader H. Moniri, Clinton E. Canal

**Affiliations:** † Department of Pharmaceutical Sciences, College of Pharmacy, 15473 Mercer University, Atlanta, Georgia 30341, United States; ‡ Department of Pharmaceutical Sciences, Jerry H. Hodge School of Pharmacy, Texas Tech University Health Sciences Center, Amarillo, Texas 79106, United States; § Department of Medicinal Chemistry, College of Pharmacy, 3463 University of Florida, Gainesville, Florida 32610, United States; ∥ Division of Therapeutics and Medical Consequences, National Institute on Drug Abuse, National Institutes of Health, Bethesda, Maryland 20892, United States; ⊥ Department of Biomedical Sciences, School of Medicine, Mercer University, Macon, Georgia 31207, United States

**Keywords:** alpha2, α2-adrenoceptor, alpha1, α1-adrenoceptor, mitragynine, clonidine

## Abstract

Mitragynine is a psychoactive alkaloid in *Mitragyna speciosa* with unique polypharmacology at G protein-coupled receptors. In addition to its well-known partial agonist activity at opioid receptors, mitragynine is an antagonist at human α_2A_-adrenoceptors (α_2A_Rs), as measured in an in vitro GTPγS G protein assay. Mitragynine’s in vitro α_2A_R antagonist pharmacology contrasts with rat behavioral pharmacology studies that suggest mitragynine behaves as an in vivo agonist at rat α_2_Rs. This study investigates this apparent discrepancy using recombinant α-adrenoceptors and a range of orthogonal signal transducers. We evaluated whether mitragynine activates any of seven Gα_i/o_ proteins coupled to α_2A_, α_2B_, and α_2C_Rs, as well as Gα_q_ and Gα_11_ coupled to α_1A_R. Additionally, we examined rat and human α_2A_R-mediated cAMP inhibition, α_2A_R-mediated β-arrestin2 recruitment, and tested α_2_R or α_1_R-mediated ERK phosphorylation in wild-type, β-arrestin 1/2 knockout, and Gα_q/11_ knockout cells. Finally, we report binding and enzyme-inhibition profiling results for mitragynine and its major metabolites, 7-Hydroxymitragynine and 9-Hydroxycorynantheidine, at 99 targets. The results did not support the hypothesis that mitragynine (or its primary metabolites) activates α_2_Rs, but, aligned with our previous GTPγS results, demonstrate that mitragynine is a low-potency, competitive α_2A_R antagonist at Gα_i1_, cAMP, and β-arrestin2 transducers. However, we show that mitragynine is a low-potency (EC_50_ ∼3 μM), partial agonist at α_1A_R-Gα_11_ and stimulates ERK phosphorylation via Gα_q/11_-coupled α_1_Rs, supporting in vivo studies that suggest mitragynine is an α_1_R agonist. Nevertheless, the agonist effects of mitragynine at α_1A_R-Gα_11_ were modest compared to clonidine, a partial agonist control that also activated all α_2_R transducers. Mitragynine’s dual α_2A_R antagonist/α_1_R partial agonist pharmacology might contribute to mitragynine’s psychostimulant-like properties.

## Graphical abstract

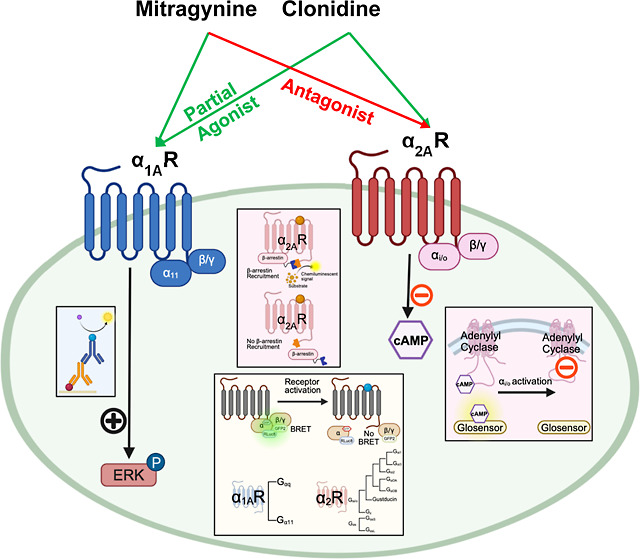

## Introduction

The leaves from *Mitragyna speciosa*, a tree native to Southeast Asia, contain more than 40 indole alkaloids,^1^ the most prevalent of which is mitragynine, accounting for up to 66% of crude alkaloid extracts.^2,3^ People in Southeast Asia have been chewing or consuming the leaves (also known as kratom) for centuries to combat work fatigue, improve mood, and alleviate physical pain.^4^ In the last two decades, kratom has also become a popular drug in the United States, where it is used recreationally or for self-medicating pain, low mood, and withdrawal symptoms associated with opioid use disorder.^5^

At μ-opioid receptors, mitragynine has low-to-moderate affinity (*K*_i_ ∼160 to 700 nM)^6–8^ and functional potency (EC_50_ ∼300 to 1000 nM)^8–10^ and behaves as a partial agonist (*E*_max_ ∼30 to 44%, relative to DAMGO or hydromorphone),^8–10^ which is thoroughly documented and consistent with its analgesic and reinforcing properties.^11,12^ Still, a growing body of literature shows mitragynine—and other kratom alkaloids—also interact with nonopioid G protein-coupled receptors (GPCRs), including serotonin and noradrenaline GPCRs,^6,13^ which may contribute to its stimulant-like effects. For example, mitragynine has low micromolar (μM) affinity at α_2_-and α_1_-adrenoceptors (αRs) expressed in vitro,^8^ and in vivo studies in mice show that idazoxan, an α_2_R and imidazoline receptor antagonist, attenuates the antinociceptive action of mitragynine.^14^ Similarly, the nonselective but potent α_2_R antagonist yohimbine and the selective α_1_R antagonist prazosin suppress the antiallodynic effects of mitragynine on chemotherapy-induced peripheral neuropathy in mice and rats.^15,16^ We have also shown in rats that mitragynine enhances the antinociceptive effects of α_2_Rs agonists (but not μ-opioid receptor agonists) and potentiates α_2_R agonist-mediated decreases in body temperature.^6^ Additionally, using a two-lever drug discrimination procedure in rats, we recently showed that the preferential α_2_R agonist lofexidine substitutes for mitragynine in mitragynine-trained subjects, and another α_2_R agonist, clonidine, partially substitutes for mitragynine. The same study also reported that the selective α_2_R antagonist atipamezole antagonizes mitragynine discrimination, indicating mitragynine and preferential α_2_R agonists exhibit similar interoceptive effects.^17^

Collectively, these studies suggest that, in vivo, mitragynine activates α_2_Rs and possibly α_1_Rs. However, in an in vitro GTPγS assay, mitragynine did not activate α_2A_Rs; rather, mitragynine exhibited competitive antagonist effects at α_2A_Rs.^17^ To reconcile the discrepancies between mitragynine’s in vitro and in vivo α_2_R activities, we executed a comprehensive study of mitragynine’s in vitro functional activity at human α_2A_, α_2B_, and α_2C_Rs. In addition to classic cAMP and β-arrestin recruitment assays, we utilized novel TRUPATH bioluminescence resonance energy transfer (BRET2) biosensors^18^ to investigate mitragynine’s ability to activate each of the seven nonvisual Gα_i/o_-protein subtypes coupled to human α_2_R subtypes. We also assessed mitragynine’s effects on rat α_2A_R-mediated cAMP inhibition in two cell lines. To address the paucity of in vitro α_1_R functional data, we also evaluated mitragynine’s ability to activate Gα_q_ and Gα_11_ coupled to human α_1A_R. Finally, we evaluated mitragynine’s influence on ERK phosphorylation in wild-type, Gα_q/11_ knockout, and β-arrestin1/2 knockout cells and tested the impact of α_1_R and α_2_R antagonism. We hypothesized that mitragynine is a biased agonist at α_2A_Rs or acts selectively at α_2B_Rs and/or α_2C_Rs, and that mitragynine is an agonist at α_1_Rs. We also considered whether mitragynine and selective αR subtype ligands might engage the same off-targets in vivo; for example, selective α_2_R ligands might engage α_1_Rs in vivo and vice versa, which would obfuscate conclusions. Finally, we report results of a comprehensive receptor, ion channel, and transporter binding and enzyme inhibition screen of mitragynine and its active metabolites, 7-Hydroxymitragynine and 9-Hydroxycorynantheidine, at 100 nM and 10 μM. The screen included tests of >70 receptors, including α-adrenoceptors, to address the possibility that mitragynine’s active metabolites contribute to the in vitro and in vivo α-adrenoceptor discrepancies of mitragynine.

## Results and Discussion

### Mitragynine Did Not Activate Human or Rat α_2A_Rs as Measured in cAMP Functional Assays

We focused on α_2A_Rs owing to their greater expression in the brain than α_2B_ and α_2C_Rs,^19–22^ and their more apparent regulation of dopamine release in the nucleus accumbens,^23^ which may contribute to reinforcing effects. Canonical α_2A_R signaling involves the activation of Gα_i_ proteins, which leads to the inhibition of adenylyl cyclase and decreased cAMP production. As shown in Figure 1A, with EC_50_ values reported in Table 1, clonidine at human α_2A_Rs decreased forskolin-stimulated cAMP production, confirming its α_2A_R agonist activity, whereas mitragynine at human α_2A_Rs did not impact forskolin-stimulated cAMP accumulation in CHO-K1 cells. Note that at concentrations above 100 nM, clonidine caused a modest increase in cAMP, resulting in a bell-shaped dose–response curve, which is consistent with the dual coupling of α_2A_Rs to Gα_i_ (inhibits cAMP production) and Gα_s_ (stimulates cAMP production) proteins at high agonist concentrations and receptor densities.^24–31^ We therefore tested the ability of clonidine and mitragynine to activate Gα_s_ proteins coupled to human α_2A_Rs using TRUPATH. Clonidine activated α_2A_R-Gα_s(short)_ with a similar potency as α_2A_R-Gα_i1_, but with ∼6-fold lower efficacy; mitragynine had no effect (Figure S1).

**Figure 1:**
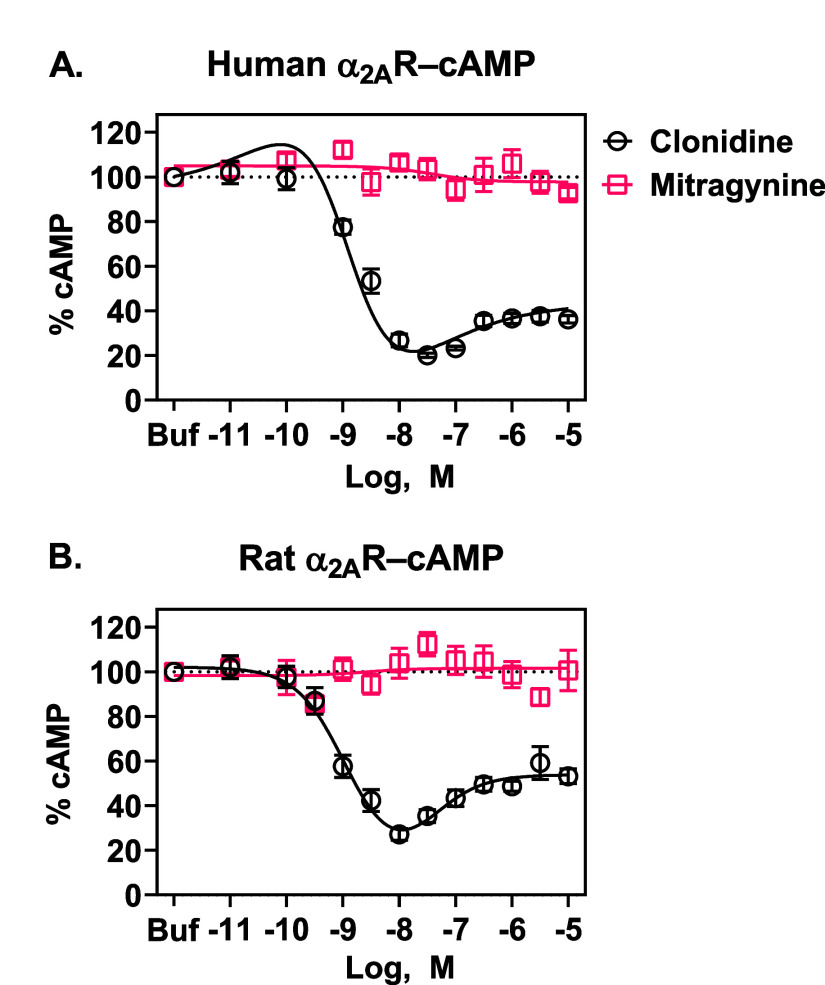
Mitragynine and clonidine activity at human (A) and rat (B) α_2A_R–cAMP in CHO-K1 cells. (A) Clonidine at human α_2A_Rs attenuated forskolin-induced cAMP production in a concentration-dependent manner, whereas mitragynine showed no significant functional effects. Note that clonidine caused an increase in cAMP production at concentrations above 100 nM, consistent with observations of a Gα_i/o_ to Gα_s_ functional switch^27–31^ (Figure S1). (B) Clonidine at rat α_2A_Rs attenuated forskolin-induced cAMP production in a concentration-dependent manner. Mitragynine showed no significant functional effects. Results were similar in rat α_2A_R-expressing HEK293 cells (Figure S2). Data show the percent of forskolin-induced cAMP accumulation. Symbols represent means ± SEMs from at least three independent assays, generating 11 and 16 technical replicates of each concentration of clonidine and mitragynine at human α_2A_R, respectively, and 10 and 11 technical replicates for clonidine and mitragynine at rat α_2A_R, respectively. Buf = buffer.

**Table 1:** Ligand Potencies as nM EC_50_ or *K*_b_ (pEC_50_ or p*A*_2_, 95% CIs) at α_2A_R Transduction Pathways

ligand	cAMP^a^	β-arrestin	αi1	αi2	αi3	αoA	αoB	αz	αgust
clonidine	h = 1.62 (8.79, 8.68–8.89) *r* = 0.935 (9.03, 9.30 to 8.56)	N.D	10.5 (7.98, 7.94–8.02)	5.62 (8.25, 8.19–8.31)	33.1 (7.48, 7.40–7.55)	2.57 (8.59, 8.55–8.62)	5.02 (8.30, 8.25–8.36)	1.74 (8.76, 8.69–8.83)	12.4 (7.91, 7.63–8.19)
UK 14, 304 (brimonidine)	N.D	2.24 (8.65, 8.44–8.86)	N.D	N.D	N.D	N.D	N.D	N.D	N.D
mitragynine	antagonist 4173 (5.380, 5.136–5.570)	antagonist 5012 (5.30, 4.98–5.59)	antagonist 5648 (5.248, 5.352–5.135)	inactive	inactive	inactive	inactive	inactive	inactive
atipamazole	N.D	antagonist 6.92 (8.16, 7.98–8.34)	N.D	N.D	N.D	N.D	N.D	N.D	N.D

a Bell-shaped curves best fit the cAMP data; shown are values derived from the first plateau. See Figure 1 h = human α_2A_R and *r* = rat α_2A_R. N.D. = not determined.

As our in vivo work suggested mitragynine activates α_2_Rs in rats, we also examined the effects of mitragynine compared to clonidine on cAMP production in CHO-K1 cells expressing rat α_2A_Rs. Clonidine was an agonist at rat α_2A_Rs (Figure 1B), and its potency at rat receptors was similar to its potency at human receptors (Table 1). Mitragynine, however, did not decrease forskolin-stimulated cAMP accumulation, showing it lacks the ability to activate the rat α_2A_R-cAMP inhibition signaling pathway (Figure 1B, Table 1). We also observed similar effects of clonidine and mitragynine in HEK293 cells expressing rat α_2A_Rs (Figure S2), supporting the validity of the observed effects across cell lines. These results suggest that interspecies variation of α_2A_Rs, or cell types expressing them, does not contribute to the in vivo and in vitro pharmacological discrepancies with mitragynine.

### Mitragynine Did Not Activate Gα_i/o_ Proteins Coupled to Human α_2A_, α_2B_, or α_2C_Rs

We next considered the possibility that mitragynine is a G protein-biased agonist at human α_2_Rs; the ability to preferentially activate G proteins that signal through cAMP-independent pathways could explain why mitragynine did not display agonistic activity in cAMP assays. For example, it is not clear whether Gα_o_ regulates cAMP production. While some studies show Gα_o_ negatively regulates adenylyl cyclases,^32^ others show it has no inhibitory effect on adenylyl cyclases.^33,34^ To assess mitragynine’s ability to activate distinct Gα_i/o_-proteins coupled to α_2_Rs, we utilized TRUPATH technology that allows for the detection of GPCR-induced Gα subunit dissociation from Gβγ subunits (Gα activation).^18^ In HEK293 cells expressing human α_2A_Rs, the Gα_i/o_ subtypes Gα_i1_, Gα_i2_, Gα_i3_, Gα_oA_, Gα_oB_, Gα_*z*_, or Gα_Gust_ fused to RLuc8, Gβ, and Gγ-GFP2, mitragynine did not activate any of the seven Gα_i/o_-protein subtypes at concentrations up to 30 μM (Figure 2). Clonidine, however, was potent and efficacious at activating each of the Gα_i/o_ proteins coupled to α_2A_Rs (Figure 2); EC_50_ values are shown in Table 1. We also screened mitragynine and clonidine at 10 μM concentrations for their ability to activate each of the Gα_i/o_ subtypes coupled to human α_2B_Rs (Figure 3) and α_2C_Rs (Figure 4). Mitragynine had no significant activity. Clonidine, however, significantly activated each of the Gα_i/o_ subtypes (*F* = 4.33 to 1054; *P* < 0.05 to 0.0001). The relatively small signal window with clonidine is likely related to its partial agonist activity at α_2_Rs;^35–37^ to verify partial agonist effects at α_2B_ and α_2C_Rs, we tested clonidine’s pharmacology at α_2B_-Gα_i2_ and α_2C_-Gα_i2_ compared to epinephrine, and observed that clonidine was a moderate potency (EC_50_ values were 61 nM at α_2B_ and 14 nM at α_2C_) partial agonist at both receptors with *E*_max_ values of ∼23–30% relative to epinephrine (EC_50_ values were 188 nM at α_2B_ and an 148 nM at α_2C_) (*N* = 1 with three technical replicates for each ligand concentration tested, figures not shown). We also tested the unlikely possibility that clonidine and mitragynine activate Gα_q_ or Gα_11_ via α_2A_Rs; we observed no effect of either drug (data not shown). Finally, we addressed the possibility that we observed no agonist effects of mitragynine at α_2_Rs, because of too short an exposure time;^38^ we extended clonidine and mitragynine’s exposure to α_2C_Rs to 30 min before adding substrate (coelenterazine) and measuring and Gα_i1_ activation via BRET, and, like the 5 min exposure, only clonidine showed agonist effects, up to 10 μM (Figure S3). These results indicate that mitragynine is not a G protein-biased agonist at α_2_Rs.

**Figure 2:**
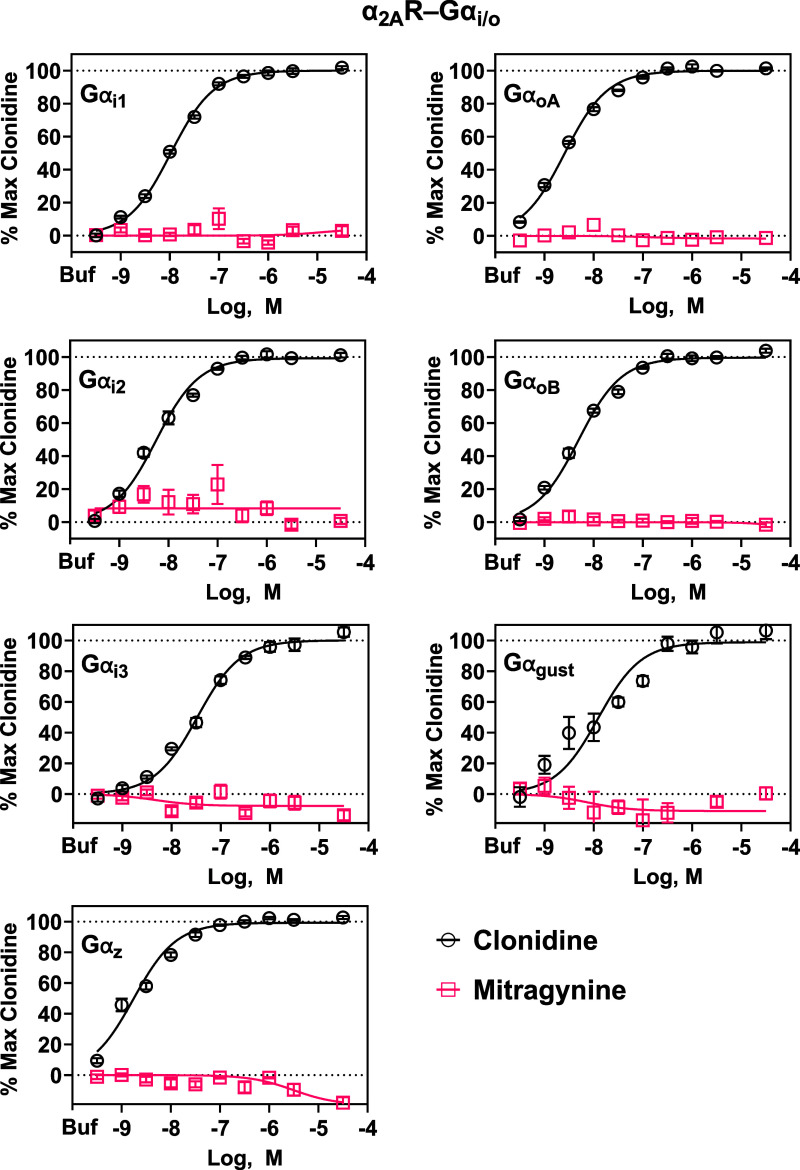
Mitragynine and clonidine activity at α_2A_R-Gα_i/o_ in HEK293 cells using TRUPATH BRET2 biosensors. Clonidine activated all Gα_i/o_ subtypes coupled to human α_2A_Rs, and mitragynine activated none. Data were normalized to the maximal response of clonidine. Each symbol represents the mean ± SEM from at least three independent assays, generating 10 technical replicates of each concentration of clonidine and mitragynine at all G proteins, except Gα_*z*_ where there were 14 technical replicates. Buf = buffer.

**Figure 3:**
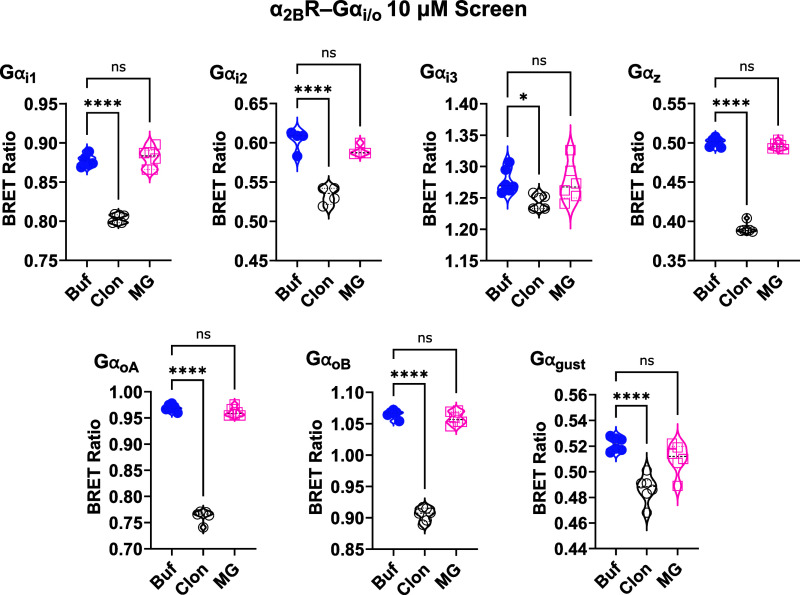
Screening of 10 μM mitragynine and clonidine activity at α_2B_R-G_i/o_ in HEK293 cells using TRUPATH BRET2 biosensors. Clonidine activated all Gα_i/o_ subtypes coupled to human α_2B_Rs, and mitragynine activated none. Technical replicates are shown as individual data points in the violin plots. *P < 0.05; ****P < 0.0001. Buf = buffer; Clon = clonidine, MG = mitragynine.

**Figure 4:**
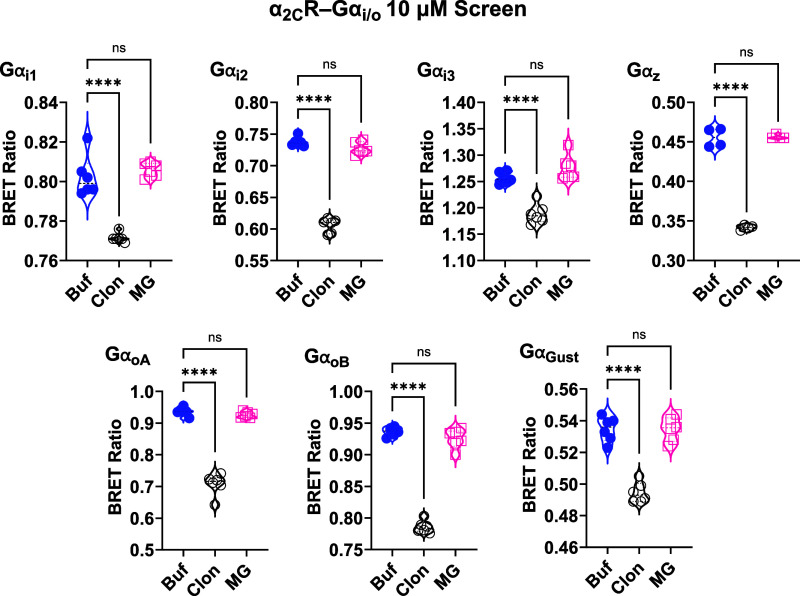
Screening of 10 μM mitragynine and clonidine activity at α_2C_R-G_i/o_ in HEK293 cells using TRUPATH BRET2 biosensors. Clonidine activated all G_i/o_ subtypes coupled to human α_2C_Rs, and mitragynine activated none. Technical replicates are shown as individual data points in the violin plots. ****P < 0.0001. Buf = buffer; Clon = clonidine, MG = mitragynine.

### Mitragynine Competitively Antagonized Clonidine-Induced Activation of Human α_2A_R-Gα_i1_ and α_2A_R-cAMP Signaling

As mitragynine did not activate α_2A_R–cAMP inhibition or α_2A_R-G protein transduction pathways, we next tested whether it behaves as an α_2A_R antagonist at them. In HEK293 cells expressing human α_2A_Rs, mitragynine caused a surmountable rightward shift in the cAMP inhibition, concentration–response curve of clonidine (Figure 5A), indicating that mitragynine acts as a competitive antagonist at human α_2A_R–cAMP; the p*A*_2_ and *K*_b_ of mitragynine, quantified using Gaddum-Schild analysis of the first plateau of the bell-shaped curve, are reported in Table 1. Similarly, in HEK293 cells expressing human α_2A_Rs with Gα_i1_-RLuc8, Gβ_3_ and Gγ_9_-GFP2 TRUPATH biosensors, mitragynine shifted the clonidine concentration–response curve to the right in a surmountable manner (Figure 5B), showing that mitragynine is a competitive antagonist at α_2A_R-Gα_i1_; the p*A*_2_ and *K*_b_ of mitragynine, quantified using Gaddum-Schild analysis, are reported in Table 1. The *K*_b_ of mitragynine at both α_2A_R–cAMP inhibition and α_2A_R–Gα_i1_ was notably similar to mitragynine’s antagonist potency at α_2A_R–GTPγS and its *K*_i_ at α_2A_R.^8,17^

**Figure 5:**
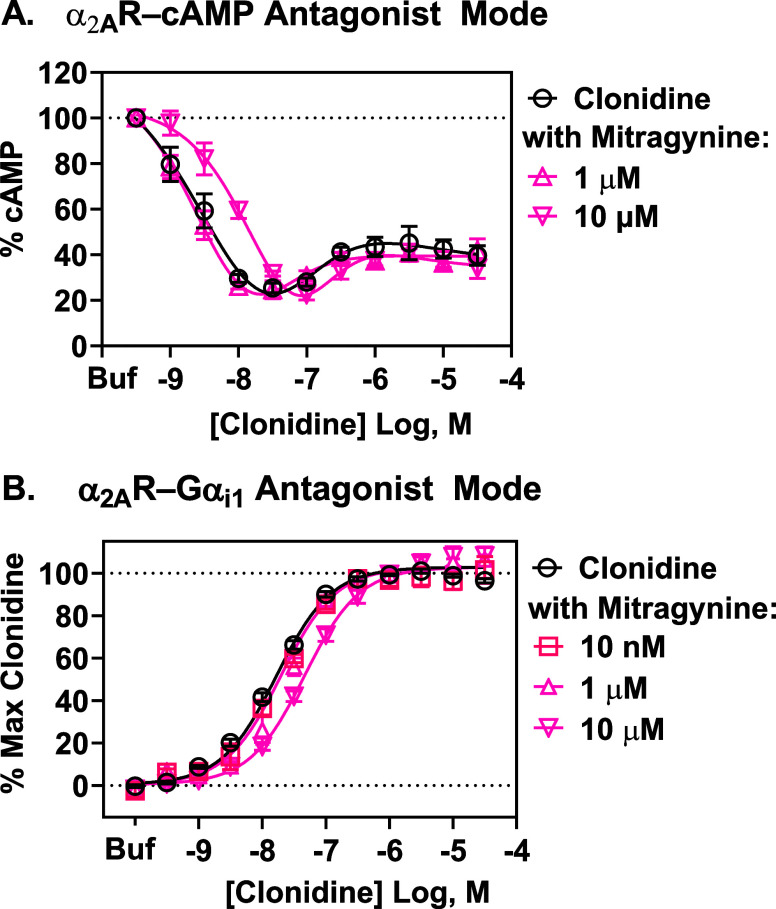
Mitragynine antagonist activity at α_2A_R-cAMP and α_2A_R-Gα_i1_. (A). In Glosensor cAMP assays, 10 μM of mitragynine produced a parallel rightward shift of the clonidine concentration–response curve in α_2A_R-expressing CHO-K1 cells. Each symbol represents the mean ± SEM from two independent assays, generating six technical replicates of each clonidine concentration, four of clonidine plus 1 μM mitragynine, and six of clonidine plus 10 μM mitragynine. Data expressed as % of forskolin-induced cAMP accumulation. (B). In TRUPATH BRET2 assays measuring Gα_i1_ coupling to α_2A_Rs in HEK293 cells, increasing concentration of mitragynine resulted in a rightward shift of the clonidine concentration–response curve without altering the *E*_max_. Data were normalized to the maximal response of clonidine. Each symbol represents the mean ± SEM from at least two independent assays, generating 23 technical replicates of each concentration of clonidine, four of clonidine plus 10 nM, four of clonidine plus 1 μM mitragynine, and 25 of clonidine plus 10 μM mitragynine. Buf = buffer.

### Mitragynine Did Not Stimulate Human α_2A_R-Mediated β-arrestin2 Recruitment, and Like G Protein and cAMP Transduction, Was an Antagonist of α_2A_R-β-arrestin2

Since mitragynine did not activate canonical α_2_R G protein transducers, we next tested if mitragynine behaves as a β-arrestin2 biased agonist at α_2A_Rs. UK-14,304, the positive control α_2A_R agonist, caused robust α_2A_R-β-arrestin2 recruitment. However, mitragynine, like the α_2A_R antagonist atipamezole, did not recruit β-arrestin2 (Figure 6A). Instead, mitragynine, like atipamezole, antagonized UK 14,304-mediated recruitment of β-arrestin2 (Figure 6B); Table 1 reports UK 14,304’s potency and mitragynine’s and atipamezole’s p*A*_2_ and *K*_b_ values; values were calculated using the Cheng–Prusoff equation. These data suggest that mitragynine is not a β-arrestin2-biased agonist at α_2A_Rs. Instead, mitragynine behaves as an antagonist at α_2A_Rs-β-arrestin2, like we observed with Gα_i1_ and cAMP. The *K*_b_ of mitragynine at α_2A_R-β-arrestin2 was similar to its *K*_b_ at α_2A_R-cAMP and α_2A_R-Gα_i1_ and aligned with mitragynine’s antagonist potency at α_2A_R-GTPγS and its *K*_i_ at α_2A_R.^8,17^

**Figure 6:**
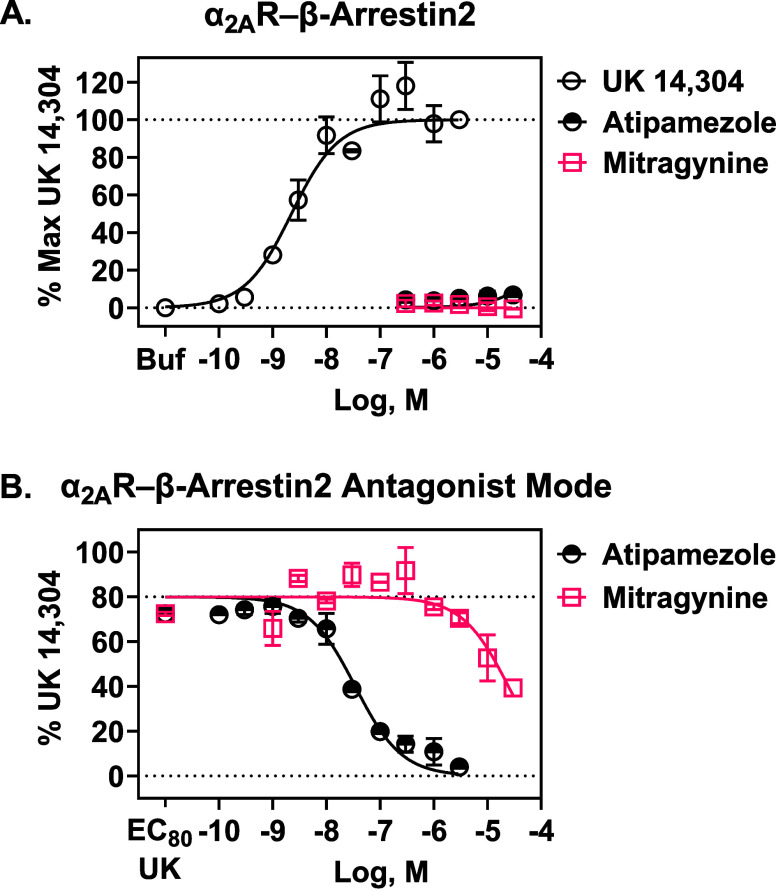
Activity at α_2A_R-β-arrestin2 in CHO-K1 cells. (A). Like the selective α_2_R antagonist, atipamezole, mitragynine did not recruit β-arrestin2 in α_2A_R-expressing cells, whereas treatment with the α_2_R agonist UK 14,304 showed reliable β-arrestin2 recruitment. B. Assays run in antagonist mode using the EC_80_ concentration of UK 14,304 showed that mitragynine was a low-potency antagonist at α_2A_Rs, relative to atipamezole. Each symbol represents the mean ± SEM from two independent assays, generating four technical replicates of each concentration of the test agents. Buf = buffer.

Based on our observations that mitragynine is not an α_2A_R agonist as measured using multiple orthogonal functional assays and is not an agonist of any α_2B_ or α_2C_R–Gα_i/o_ transducers, but instead behaves as an α_2A_R antagonist at all transducers examined, we can only speculate on what mechanism(s) led to clonidine (and lofexidine) substituting for mitragynine and atipamezole antagonizing mitragynine in rat drug discrimination assays.^17^ One possibility is the off-target effects of ligand probes in vivo. Although clonidine is a selective α_2_R agonist, its binding selectivity is only about 10-fold over α_1_Rs,^39^ and it stimulates α_1_R-mediated physiological responses,^40^ suggesting clonidine’s in vivo interoceptive cue might involve activation of α_1_Rs. Future in vivo studies that test selective α_2_R ligands at doses shown to engage brain α_2_Rs, with no engagement of α_1_R, could unravel the possibility that mitragynine’s in vivo interoceptive cue is mediated by α_1_Rs, which is mimicked by clonidine. It is also possible that focusing on end points proximal to the receptor (e.g., G proteins), which lack appreciable stimulus-response coupling, could mask low efficacy α_2_-adrenoceptor partial agonism only apparent in other downstream end points, e.g., gene transcription changes.

### Mitragynine Was a Low-Potency α_1A_R-Gα_11_ Partial Agonist

As clonidine has appreciable affinity and agonist potency at α_1_Rs,^39,41^ we next evaluated mitragynine’s activity at α_1A_Rs, compared to clonidine. We focused on α_1A_Rs owing to their relatively dense expression in neural systems controlling arousal, their regulation of brain activity associated with arousal, and their regulation of serotonergic neuron activity, which align with mitragynine’s psychostimulant effects.^13,42–46^ We used TRUPATH to measure the activation of Gα_q_ and Gα_11_ coupled to α_1A_Rs. Figure 7A and Table 2 show that mitragynine was a partial agonist at α_1A_R-Gα_11_, relative to clonidine. Encouragingly, mitragynine’s EC_50_ at α_1A_R-Gα_11_ (Table 2) was in line with its affinity (*K*_i_) at α_1A_Rs.^8^ Clonidine, but not mitragynine, also activated α_1A_R-Gα_q_, shown in Figure 7B and Table 2; there was no significant difference between mitragynine’s efficacy at α_1A_R-Gα_q_ compared to buffer (*P* > 0.05).

**Figure 7:**
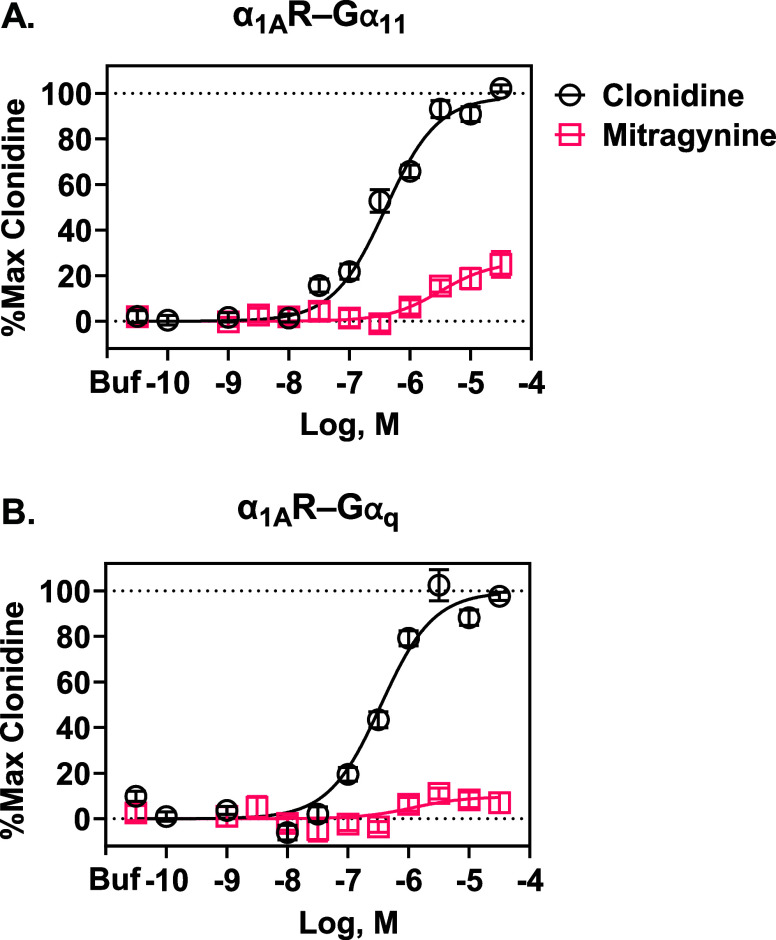
Mitragynine and clonidine activity at human α_1A_R-Gα_11_ and α_1A_R-Gα_q_ expressed in HEK293 cells. (A). Clonidine and mitragynine were agonists at α_1A_R-Gα_11_, with mitragynine having lower efficacy. Data were normalized to the maximal response of clonidine. Each symbol represents the mean ± SEM from at least 10 independent assays, generating 35 technical replicates of each clonidine concentration and 49 of mitragynine. Note clonidine (and mitragynine) was also a partial agonist relative to epinephrine (30 technical replicates), as was lofexidine (30 technical replicates); see Table 2. (B). Clonidine was an agonist at α_1A_R-Gα_q_, whereas mitragynine showed no significant functional effects. Data were normalized to the maximal response of clonidine. Each symbol represents the mean ± SEM from five independent assays, generating 15 technical replicates of each clonidine and mitragynine concentration. Lofexidine was also an agonist at α_1A_R-Gα_q_ (*N* = 2, 6 technical replicates); see Table 2. Buf = buffer.

**Table 2:** Ligand Potencies as nM EC_50_ (pEC_50_, 95% CIs) and *E*_max_ Values as Percent of Clonidine’s Maximum Response at α_1A_R Transducers^a^

ligand	α11	αq
clonidine	389 (6.41, 6.31–6.50) *E*_max_ = 100%	347 (6.47, 6.36–6.55) *E*_max_ = 100%
lofexidine	112 (6.95, 6.80–7.10) *E*_max_ = 76%	95.5 (7.02, 6.83–7.20) *E*_max_ = 84%
epinephrine	129 (6.89, 6.80–6.98) *E*_max_ = 279%	N.D
mitragynine	3162 (5.50, 4.91–6.04) *E*_max_ = 27%	inactive

a Note the higher efficacy of epinephrine at α_1A_-Gα_11_ relative to all other test agents, indicating their partial agonist effects.

Notably, clonidine itself was a partial agonist relative to epinephrine at both α_1A_R-Gα_11_ and α_1A_R-Gα_11_, as was lofexidine. Table 2 reports the potency and efficacy values of mitragynine, clonidine, lofexidine, and epinephrine at α_1A_Rs. The shared partial agonist activity of mitragynine, clonidine, and lofexidine at α_1A_R-Gα_11_ might explain clonidine’s and lofexidine’s ability to substitute for mitragynine in drug discrimination assays.^17^ The agonist effects of mitragynine at α_1A_R-Gα_11_ might also explain why prazosin blocks mitragynine’s antinociceptive effects.^15,16^

### Mitragynine Stimulated pERK via α_1_Rs and Gα_q/11_

Next, we examined the effect of mitragynine compared to clonidine on ERK phosphorylation (pERK) in HEK293 cells. We probed α_2_R-dependent effects by cotreating cells with 100 nM atipamezole and α_1_R-dependent effects by cotreating cells with 100 nM prazosin. In vitro, atipamezole has double-digit nM affinity (*K*_d_) at each of the human α_2_Rs, 1000 nM or lower affinity at each of the α_1_Rs, and >30,000 nM affinity at β_1_ and β_2_ adrenoceptors.^41,47^ Prazosin has single-digit nM or higher affinity (*K*_d_) at human α_1_Rs, ∼300 nM or lower affinity at each of the α_2_Rs, and ∼8000 nM or lower affinity at β_1_ and β_2_ adrenoceptors.^41,47^ We also probed the dependence of treatment effects on β-arrestin1/2 and Gα_q/11_ by examining effects in β-arrestin1/2 and Gα_q/11_ knockout HEK293 cells. The selective β-adrenoceptor agonist, isoproterenol, was used as a positive control for pERK, as isoproterenol robustly stimulates pERK in nontransfected HEK293 cells that endogenously express β-adrenoceptors.^48–51^ We initially tested HEK293 cells transfected with human α_2A_Rs. As shown in Figure 8A, 5 min treatment with 1 μM isoproterenol (ISO) and 1 μM clonidine (CLON) significantly increased pERK relative to buffer (*P* < 0.01), and 5 min treatment with 10 μM mitragynine also significantly increased pERK (*P* < 0.05). As GPCR-mediated pERK is sensitive to receptor levels,^48–51^ we next tested treatment effects in nontransfected HEK293 cells. In these cells, as shown in Figure 8B, ISO and CLON again stimulated pERK (*P* < 0.01 and *P* < 0.05), and 1, 5, 15, and 30 min treatment with 1 or 10 μM mitragynine also significantly increased pERK (*P* < 0.01 at all time points but 15 min, *P* < 0.05). In addition to β-adrenoceptors, HEK293 cells also endogenously express functional α_2_ and α_1_Rs,^52^ with α_1B_R mRNA expression reportedly much higher than the expression of α_2_R subtypes.^53^ GPCR overexpression in HEK293 cells dramatically increases agonist efficacy at GPCR-pERK,^48^ yet we did not observe substantial differences in treatment-induced pERK in α_2_R-transfected compared to nontransfected HEK293 cells (Figure 8A,B). Also, the selective α_2_R agonist, UK14,304—which, compared to clonidine, is more selective and efficacious at α_2_Rs than α_1_R^41,54^ —did not increase pERK, but the selective α_1_R agonist, phenylephrine, induced robust increases in pERK in nontransfected HEK293.^52^ These observations gave us the initial clues that α_2_R’s do not stimulate pERK in our HEK293 cells.

**Figure 8:**
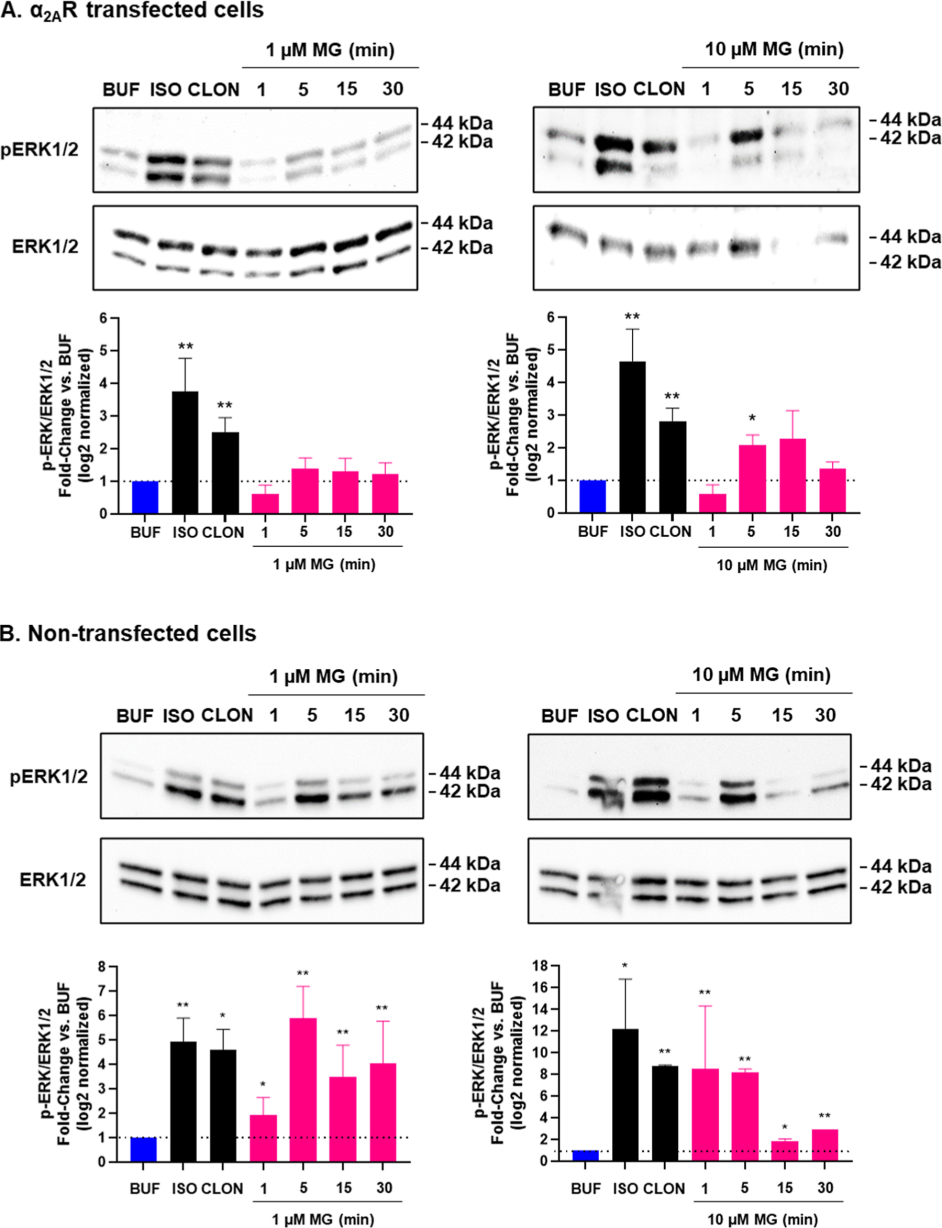
Effects of clonidine and mitragynine on ERK phosphorylation in α_2A_R-transfected and nontransfected HEK293 cells. Clonidine (CLON, 1 μM, 5 min) and mitragynine (MG) stimulated pERK in both cells, like isoproterenol (ISO, 1 μM, 5 min). (A). Tests of α_2A_R-transfected cells included five independent assays for all groups, except MG 30 min (*N* = 4). (B). Screening of nontransfected cells included two independent assays for all groups; based on the test agents stimulating pERK in nontransfected cells, they were used in subsequent experiments, which replicated the effects of the test agents (Figures 9 and 10). All bars show mean ± SEM values. BUF = buffer. *P < 0.05, **P < 0.01. Buf = buffer.

Given the robust treatment-induced pERK effects in nontransfected cells, we tested nontransfected cells for the remaining experiments. We treated cells with mitragynine for 5 min in all subsequent pERK experiments, because in our initial studies, mitragynine consistently increased pERK with this treatment exposure time. We examined treatment effects in CRISPR scrambled and Gα_q/11_ knockout (Figure 9A) and CRISPR scrambled and β-arrestin1/2 knockout (Figure 9B) HEK293 cells. The pERK-stimulating effects of CLON were abolished in Gα_q/11_ knockout cells (*P* < 0.01), as were the pERK-stimulating effects of 1 μM mitragynine (*P* < 0.01). We did not observe differences in pERK in β-arrestin1/2 knockout cells. These observations demonstrated that CLON and mitragynine stimulate pERK through Gα_q/11_ and aligned with our observations that clonidine activates Gα_q_ and Gα_11_ coupled to α_1A_Rs while mitragynine activates Gα_11_ coupled to α_1A_Rs.

**Figure 9:**
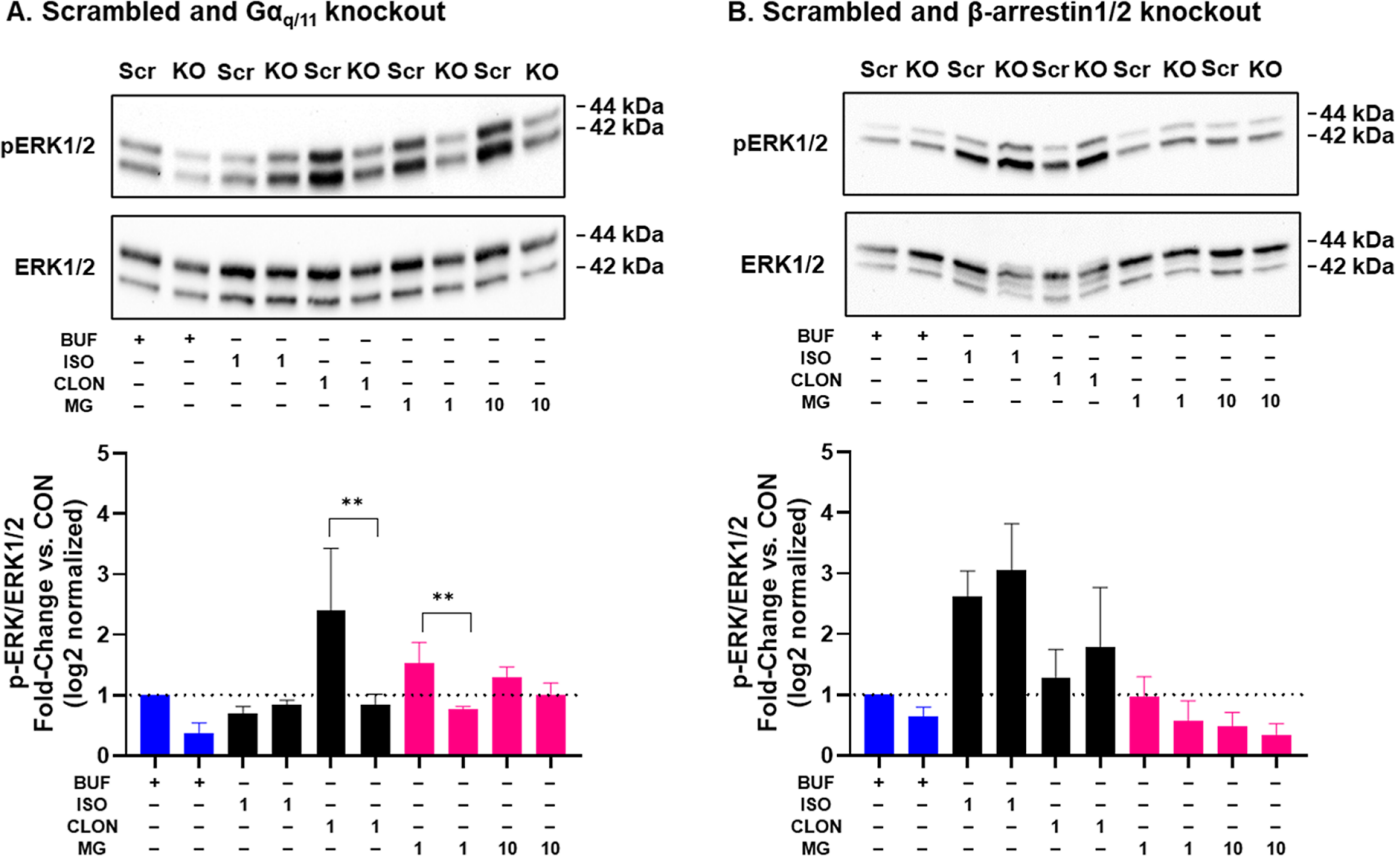
Effects of clonidine and mitragynine on ERK phosphorylation in Gα_q/11_ and β-arrestin1/2 CRISPR knockout HEK293 cells. (A). The pERK-stimulating effect of 1 μM clonidine and mitragynine was abolished in Gα_q/11_ knockout (KO) cells. (B). There were no effects of β-arrestin 1/2 KO compared to scrambled (Scr) controls. All drug exposures were for 5 min. Graphical data were generated from the results of three independent assays. **P < 0.01. Statistical tests compared the effects of the knockouts only. Buf = buffer. ISO = isoproterenol. CLON = clonidine. MG = mitragynine. One and 10 = 1 and 10 μM.

Finally, as shown in Figure 10A, cotreatment of HEK293 cells with 100 nM prazosin abolished the pERK-stimulating effects of CLON (*P* < 0.01) and 1 μM mitragynine (*P* < 0.01). Whereas, as shown in Figure 10B, cotreatment of HEK293 cells with 100 nM atipamezole did not affect the pERK-stimulating effects of CLON or mitragynine. The pERK-stimulating effects of ISO were not affected by prazosin or atipamezole, which further validated that the 100 nM test doses selectively engaged α_1_ and α_2_Rs, respectively. These observations showed that CLON and mitragynine stimulate pERK through α_1_Rs, not α_2_Rs. In support of these conclusions, several prior reports show that α_1_R activation stimulates pERK,^55–57^ and a recent report shows that clonidine stimulates α_1A_ and α_1B_R-pERK with an EC_50_ below 100 nM^41^.

**Figure 10:**
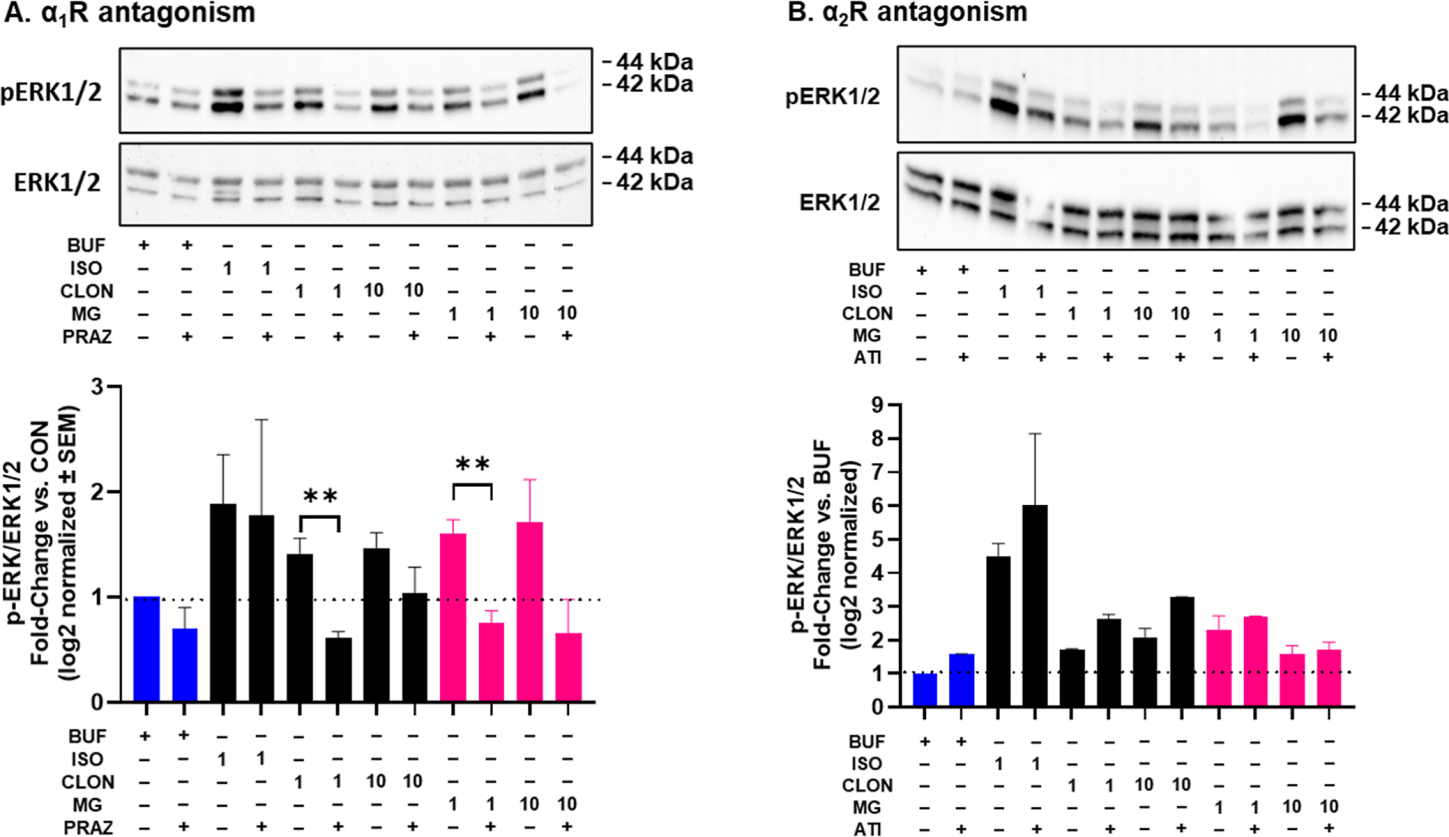
Effects of selective α_2_R (100 nM atipamezole (ATI), 10 min pretreatment) and α_1_R (100 nM prazosin, 10 min pretreatment) antagonism on ligand-stimulated pERK in HEK293 cells. Statistical tests compared the effects of the antagonists only. (A). There was no effect of atipamezole. (B). Pretreatment with prazosin significantly attenuated clonidine and MG-stimulated ERK activity. Graphical data were generated from the results of three independent assays. Statistical tests compared the effects of the antagonists only. BUF = buffer. ISO = isoproterenol. CLON = clonidine. MG = mitragynine. PRAZ = prazosin. ATI = atipamezole.

### The Major Metabolite of Mitragynine, 7-Hydroxymitragyine, Did Not Have Appreciable Affinity at α_2_-or α_1_-Adrenoceptors

Since the discrepancies between mitragynine’s in vitro (antagonist) and in vivo (agonist-like) α_2_R pharmacology might be explained by active metabolites, we assessed the binding of the major mitragynine metabolite, 7-Hydroxymitragynine,^58^ at α-adrenoceptors and other receptors, ion channels, and transporters, and profiled its enzyme inhibition activity in a 100 nM and 10 μM screen; activity at 99 targets was probed (Supplemental Figure S4). We previously reported the opioid receptor, serotonin receptor, and α-adrenoceptor results,^8,13^ and reiterate that neither 100 nM nor 10 μM 7-Hydroxymitragynine effectively bound α_2A_ or α_2B_Rs, e.g., 10 μM 7-Hydroxymitragynine inhibited less than 15% of specific radioligand binding. Similar to its lack of affinity at α_2_-adrenoceptors, 10 μM 7-Hydroxymitragynine inhibited less than 15% of specific radioligand binding at α_1A_, α_1B_, and α_1D_Rs. Of all proteins screened, 10 μM 7-Hydroxymitragynine bound significantly—more than 50% inhibition of radioligand binding—to only μ-, δ-, and κ-opioid receptors, illustrating its remarkable selectivity at opioid receptors. Ten μM 7-Hydroxymitragynine also did not inhibit the activity of any enzyme tested. These data indicate that mitragynine’s major metabolite, 7-Hydroxymitragynine, does not account for the discrepancies in mitragynine’s in vitro versus in vivo α_2_-or α_1_-adrenoceptor pharmacology.

### The Major Metabolite of Mitragynine, 9-Hydroxycorynantheidine, did Not Have Appreciable Affinity at α_2_-Adrenoceptors But Had Significant Binding at α_1_-Adrenoceptors

In addition to 7-Hydroxymitragynine, we profiled the binding and enzyme inhibition of the major mitragynine metabolite, 9-Hydroxycorynantheidine^59,60^ (Supplemental Figure S5). We previously reported the opioid receptor, serotonin receptor, and α-adrenoceptor results,^8,13^ and reiterate that neither 100 nM nor 10 μM 9-Hydroxycorynantheidine effectively bound α_2A_ or α_2B_Rs, e.g., 9-Hydroxycorynantheidine inhibited less than 25% of specific radioligand binding at 10 μM. However, in contrast to its unappreciable affinity at α_2_-adrenoceptors, 10 μM 9-Hydroxycorynantheidine inhibited more than 60% specific radioligand binding at α_1A_ and α_1D_Rs, suggesting that 9-Hydroxycorynantheidine may contribute to the purported in vivo α_1_-adrenoceptor activity of mitragynine. At 10 μM, 9-Hydroxycorynantheidine hit other targets, which were not previously reported. It bound significantly at the serotonin transporter (∼58% inhibition) and voltage-gated sodium channels (∼57% inhibition), and it inhibited COX2 (>70%), which, if engaged in vivo, could contribute to mitragynine’s analgesic and cardiac effects.

### Binding and Enzyme Inhibition Profile of Mitragynine

The binding and enzyme inhibition of 100 nM and 10 μM mitragynine were also assessed (Supplemental Figure S6). We previously reported the opioid receptor, serotonin receptor, and α-adrenoceptor results.^8,13^ Compared to its metabolites 7-Hydroxymitragynine and 9-Hydroxycorynantheidine, mitragynine had unique pharmacology, which was not previously reported. Ten μM mitragynine hit β_2_-adrenoceptors (>60% inhibition of specific radioligand binding), D_1_-(∼73% inhibition), D_2_-(∼70% inhibition), and D_3_-dopamine receptors (∼57% inhibition), the serotonin transporter (∼51%), hERG potassium channels (∼51%), and voltage-gated sodium channels (∼71% inhibition).

## Conclusion and Future Direction

Here we report the results of a comprehensive study of mitragynine’s α-adrenoceptor pharmacology and show that mitragynine has low μM potency, α_2A_R antagonist and α_1A_R-G_11_ partial agonist properties. The following rationale supports the conclusion that mitragynine’s α-adrenoceptor pharmacology is physiologically relevant. In vitro, mitragynine is well-appreciated to be a low-to-moderate potency partial agonist at μ-opioid receptors,^6–10^ and its potency at μ-opioid receptors is only ∼3–13-fold higher than its potency at α-adrenoceptors. In vivo, mitragynine has low-potency, μ-opioid receptor-dependent analgesic effects. For example, mitragynine’s analgesic ED_50_ in mice in the hot plate assay after PO administration is 135.5 mg/kg (340 μmol/kg), and its analgesic effects are blocked by naltrexone, a μ-opioid receptor antagonist.^61^ At a PO dose close to its ED_50_ (165 mg/kg), brain concentrations of mitragynine in male mice peak at approximately 35 μg/g of wet brain tissue, or ∼88 μM.^61^ Although free concentrations and occupancies at brain receptor targets were not determined, 88 μM of mitragynine in the brain—which is > 20-fold higher than mitragynine’s binding affinity and functional potencies at α-adrenoceptors—suggests it likely targets α-adrenoceptors at active doses. Mitragynine’s α-adrenoceptor pharmacology as an α_2_-adrenoceptor antagonist/α_1_-adrenoceptor partial agonist would likely translate to sympathomimetic activity and stimulate arousal upon target engagement, thereby contributing to mitragynine’s psychostimulant-like effects. It also—along with mitragynine’s direct interaction with specific serotonin GPCRs^13^—may contribute to mitragynine’s serotonin-stimulating-like effects, as α_2_Rs and α_1_Rs regulate serotonin neuron activity.^62^

Despite the extensive investigation of mitragynine’s in vitro α_2_-adrenoceptor pharmacology, our observations conflict with results from our and others’ in vivo observations that suggest mitragynine acts as an α_2_-adrenoceptor agonist. Such discrepancy is not uncommon, as mitragynine was also found to be a competitive antagonist at mouse μ-opioid receptors in a cell-based assay,^63^ conflicting with the antinociceptive actions of mitragynine in rodent models, which selective μ-opioid receptor antagonists block. Though interassay variation could be the general explanation for the discrepancies, other possible explanations exist. The multiple locations where α_2_-adrenoceptors are expressed in the brain, and the different, sometimes opposing functions—as autoreceptors on adrenergic terminals, as heteroreceptors on glutamatergic and GABAergic terminals, and postsynaptically—render in vitro and in vivo comparisons challenging.^64^ Investigations of apomorphine provide a suitable analogy. At high doses, apomorphine directly stimulates postsynaptic dopamine D_2_ receptors; at lower doses, however, apomorphine selectively binds to presynaptic autoreceptors and inhibits the synthesis and release of dopamine, thus acting as an indirect D_2_ receptor antagonist.^65–67^ In the context of this study, mitragynine may inhibit a distinct population of α_2_-adrenoceptors, and inhibition of this population may result in the same physiological outcomes as activation of another population, stimulated by the α_2_-adrenoceptor agonists lofexidine and clonidine.

Mitragynine may also enhance α_2_-adrenoceptor transduction through direct action on effectors downstream of α_2A_Rs. In axon terminals, activation of α_2A_Rs triggers dissociation of Gα_i/o_ and βγ subunits. The released Gβγ subunits bind voltage-gated N-type Ca^2+^ channels, reducing Ca^2+^ currents and neurotransmitter release. If mitragynine directly inhibited these Ca^2+^ currents, it could achieve the same neuronal outcomes as activating α_2A_Rs. Indeed, a previous study showed that mitragynine blocks Ca^2+^ channel currents in neuroblastoma cells.^68^ However, they were from T- and L-type Ca^2+^ channels, and our observations show mitragynine has no appreciable affinity at L- or N-type Ca^2+^ channels in rat cerebral cortex (Supplementary Figure S6); we did not assess its activity at T-type Ca^2+^ channels. Studies of the interaction between mitragynine and calcium channels are scarce, so further research is needed to test this hypothesis and resolve conflicts between mitragynine’s activity at α_2_-and α_1A_-adrenoceptors, because the latter would predictably increase Ca^2+^ flux when activated. Still, experiments evaluating the activity of mitragynine at α_1B_ and α_1D_R subtypes are warranted.

Another potential explanation for the conflicting in vivo and in vitro observations regarding α_2_-adrenoceptors stems from the fact that the α_2A_R ligands used in animal studies, namely clonidine, lofexidine, idazoxan, yohimbine, and atipamezole, possess an imidazoline ring moiety and hence likely interact with imidazoline receptors (IRs). The two most well-understood IRs are imidazoline 1 receptors (I_1_Rs) and imidazoline 2 receptors (I_2_Rs). Importantly, agmatine, a putative endogenous ligand of I_1_Rs, has been shown to potentiate the analgesic effects of morphine and to reduce opioid dependence and withdrawal, effects akin to the observed effects of mitragynine. Also, in post-mortem prefrontal cortex from long-term opioid and mixed opioid/cocaine users, an upregulation of I_1_Rs was found in the membrane-associated protein fraction, suggesting recruitment of I_1_Rs to membranes in opioid dependence.^69–71^ I_2_Rs have also been demonstrated to mediate analgesic effects in various animal models of chronic pain.^72,73^ Furthermore, I_2_Rs agonists have been shown to produce discriminative stimulus effects.^74^ Taken together, though no study has examined the pharmacology of mitragynine at I_1_ and I_2_Rs, the discriminative stimulus effects and (part of) the antinociceptive effects produced by mitragynine and bona fide α_2_-adrenoceptor agonists could be mediated by them.

Finally, there is ample evidence that α-adrenoceptor signaling pathways interact with opioid receptor-mediated pain modulation, and that α-adrenoceptor antagonists and agonists can influence μ-opioid receptor effects indirectly by altering the excitability of key neurons in pain pathways.^75,76^ Although this does not explain the specific in vitro versus in vivo α-adrenoceptor pharmacology discrepancies, it highlights the complex nature of in vivo mechanisms that impact behavior.

## Methods and Materials

### Compounds

Mitragynine hydrochloride (purity ≥98%) was synthesized from mitragynine-free base (purity ≥98%), which was isolated and purified from the kratom alkaloids rich extract as previously described,^77^ and its structure was confirmed by ^1^H NMR, ^13^C NMR, and HRMS using Bruker model AMX 500 and Avance NEO 600 NMR spectrometers operating at 500 and 600 MHz in ^1^H and 126 and 151 MHz in ^13^C, respectively. HRMS and purity (≥95%) were determined using an Agilent 1290 Infinity series ultraperformance liquid chromatography (UPLC) system and a quadrupole-time-of-flight (QTOF) Agilent 6540 mass spectrometer. We previously reported spectra data for (−)-mitragynine hydrochloride.^8^ Clonidine hydrochloride was obtained from Cayman Chemical. Lofexidine hydrochloride was obtained from Alfa Aesar. UK 14,304 (brimonidine) and (±)-Epinephrine hydrochloride were obtained from Sigma-Aldrich. Isoproterenol hydrochloride and atipamezole hydrochloride were obtained from TCI America. Prazosin hydrochloride was obtained from Tocris. All compounds were dissolved in either dimethyl sulfoxide or Milli-Q water before being diluted and tested in an assay buffer.

### cAMP Inhibition

The GloSensor cAMP Assay (Promega Corporation, Madison, WI) was used to measure intracellular cAMP according to the manufacturer’s protocol. Briefly, Chinese hamster ovary CHO-K1 cells (*P* < 20 from a procured stock, CCL-61, ATCC, Manassas, VA, USA) were grown in medium containing 10% fetal bovine serum (FBS, MT35010CV, Corning, NY) and Dulbecco’s Modified Eagle’s Medium (DMEM, BE12-604Q, Lonza, Switzerland) in 10 cm cell culture plates. At 80% confluency, cells were transfected with 5 μg of human α_2A_R cDNA (cloned into pcDNA 3.1+ plasmid, cDNA Resource Center, Bloomsburg, PA) or rat α_2A_R cDNA (cloned into pCMV6-Entry vector, OriGene Global, Rockville, MD) and 1 μg of 22F GloSensor plasmid cDNA (Promega) using polyethylenimine (PEI), molecular weight ∼40,000 g/mol (Polysciences Inc., Warrington, PA) based on established methods. Twenty-4 h after transfection, cells were plated in medium containing 10% FBS (MT35010CV, Corning, NY) and DMEM (BE12-604Q, Lonza, Switzerland) in white, opaque 96-well plates (PerkinElmer, Waltham, MA) at a density of 1.5 × 10^5^ cells/100 μL. They were then incubated overnight at 37 °C, 5% CO_2_, 95% humidity. The next day, media were aspirated, and cells were incubated with 90 μL of assay buffer (1× Hank’s Balanced Salt Solution (HBSS) plus 20 mM 4-(2-hydroxyethyl)-1-piperazineethanesulfonic acid (HEPES)) and 1% GloSensor substrate for 2 h in the dark at room temperature. Cells were then treated for 30 min (min) with 10 μL of test compounds dissolved in assay buffer before incubation with 11 μL of 10 μM (final concentration) of forskolin in the dark at room temperature. Luminescence was measured with a Microbeta2Microplate Counter (PerkinElmer, Waltham, MA). The same assays were also performed with human embryonic kidney HEK293 cells (*P* < 20, from a procured stock, CRL-1573, ATCC, Manassas, VA) transfected with rat α_2A_Rs to assess potential cell species-dependent differences in functional effects.

### β-arrestin2 Recruitment

The PathHunter eXpress ADRA2A assay (Eurofins-DiscoverX, Fremont, CA) was used to determine human α_2A_R-mediated β-arrestin2 recruitment utilizing the manufacturer’s protocol. Briefly, α_2A_R-expressing, β-arrestin2-tagged CHO-K1 cells from the kit were plated into a 96-well assay plate with AssayComplete Cell Plating Reagent and incubated for 48 h at 37 °C, 5% CO_2_, 95% humidity at a density of 8.3 × 10^3^ cells/100 μL. Cells were then treated with 10 μL of test compounds and reincubated as noted for 90 min. The addition of 55 μL detection solution followed this, and the assay plate was incubated for 1 h, this time at room temperature in the dark. Luminescence was then measured using a Microbeta2 microplate counter (Revvity, Waltham, MA) and normalized relative to the maximum stimulation by the α_2_R agonist UK 14,304. To test for antagonism, varying concentrations of 5 μL test compounds were coincubated with an EC_80_ concentration (0.01 μM) of 5 μL UK 14,304.

### Gα Subunit Activation

We measured Gα subunit activation with TRUPATH BRET2 biosensors according to the original protocol.^18^ HEK293 cells (*P* < 20) were transfected at 80% confluency in 10 cm cell culture plates with a 1:1:1:1 cDNA ratio of receptor: Gα-RLuc8: Gβ: Gγ-GFP2 (750 ng per construct) using PEI. β_3_ and γ_8_ were transfected with Gα_i2_, _oA_, and _oB_; β_3_ and γ_9_ were transfected with Gα_i1_, _i3_, and α_q_; β_3_ and γ_1_ were transfected with Gα_*z*_ and _gustducin_ (_gust_); β_3_ and γ_13_ was transfected with Gα_11_ based on.^18^ Forty-8 h after transfection, cells were washed three times with assay buffer (140 mM NaCl, 2.7 mM KCl, 1 mM MgCl_2_, 1 mM CaCl_2_, 0.37 mM NaH_2_PO_4_, 24 mM NaHCO_3_, 25 mM HEPES, 0.1% Glucose, pH 7.4) before plating in white, opaque 96-well plates (PerkinElmer, Waltham, MA) in assay buffer at a density of 2.5 to 3.5 × 10^4^. After 1 h of incubation at room temperature, cells were treated with 50 μL of 5 μM (final concentration) freshly prepared coelenterazine 400a (Nanolight Technologies) for 5 min in the dark, followed by 10 μL of compounds in assay buffer for an additional 5 min. Plates were read by a Mithras LB 940 Multimode Microplate Reader (Berthold Technologies, Bad Wildbad, Germany) with 410 nm (RLuc8-coelenterazine 400a) and 515 nm (GFP2) emission filters, at a counting time of 1 s per well. BRET2 was calculated as the ratio of GFP2 to RLuc8 emission. Full dose–response tests of ligands at human α_2A_R coupled to each Gα_i/o_ protein were conducted. At human α_2B_Rs and α_2C_Rs, we screened ligands at 10 μM; as we observed no activation of α_2B_Rs or α_2C_Rs by mitragynine, we did not further evaluate its functional effects at them. Full dose–response tests of ligands at human α_1A_R coupled to Gα_q_ and Gα_11_ were also conducted.

### ERK Phosphorylation

Extracellular signal-regulated kinase 1/2 (ERK) phosphorylation was assessed as previously described.^78,79^ Briefly, α_2A_R transfected (as described above) or nontransfected HEK293 cells (*P* < 20) were plated at 1.2 × 10^6^ cells in 2 mL of media on collagen-coated six-well plates. Cells were serum-starved for 75 min and treated with ligands in 1 mL of fresh DMEM in the absence of serum as described in the figure legends. Cells were washed three times with ice-cold phosphate-buffered saline, lysed on ice in Radio-Immunoprecipitation Assay buffer, centrifuged to clarify the supernatant, normalized to protein concentration, and boiled in Laemmli sample buffer. Following sodium dodecyl sulfate–polyacrylamide gel electrophoresis, gels were transferred, blocked in 5% BSA and incubated with 1:1000 rabbit phospho-ERK1/2 (#9101, Cell Signaling Technology, Danvers, MA) and imaged with enhanced chemiluminescence (ECL). Blots were stripped and reprobed with rabbit ERK1/2 (#9102, Cell Signaling Technology) antibody and imaged with ECL to assess protein loading.

### Radioligand Binding and Enzyme Inhibition

Eurofins Cerep conducted radioligand binding and enzyme inhibition assays to elucidate the in vitro profiles of two active mitragynine metabolites, 7-Hydroxymitragynine and 9-Hydroxycorynantheidine, as well as mitragynine at 99 targets (see Supplementary Figures S4–S6). The compounds were screened at each target once, in duplicate, at 100 nM and 10 μM. In each experiment, a reference compound with known high affinity at the target (see table in Supplementary Figure S4) was tested concurrently with mitragynine, 7-Hydroxymitragynine, or 9-Hydroxycorynantheidine to assess assay reliability; the reference was tested at several concentrations for *K*_i_ (binding) or IC_50_ (enzyme inhibition) value determination, and the data were compared with historical values determined at Eurofins Cerep. The assay was valid if the reliability criteria, *K*_i_ or IC_50_ values within 1/2 Log of historical values, were met. Radioligand binding assay methods were as previously described with only minor modifications^13,80^ (see table in Supplementary Figure S4 for radioligands, protein sources, and assay test conditions). Briefly, cell membrane homogenates were incubated with either an antagonist or agonist radioligand in the absence or presence of the test compound in a buffer. A specific unlabeled agonist or antagonist at the target determined nonspecific binding. Following incubation, the samples were filtered rapidly under vacuum through glass fiber filters presoaked in a buffer and rinsed several times with an ice-cold buffer using a 48-sample or 96-sample cell harvester. Using a scintillation cocktail, the filters were counted for radioactivity in a scintillation counter. Compound binding was calculated as a percent inhibition of the binding of the radioactively labeled ligand specific for each target. Preparatory methods for enzyme inhibition assays were similar to radioligand binding assays. These assays involved using a known substrate, stimulus, and/or tracer, and detection methods included LANCE, fluorimetry, photometry, luminescence, or scintillation counting (see table in Supplementary Figure S4). Results of the screening assays showing an inhibition or stimulation higher than 50% were designated “hits” and considered to represent significant effects of the test compounds.

### Data Analyses

All data were analyzed using GraphPad 9.5 (San Diego, CA, USA). EC_50_, *E*_max_, p*A*_2_, and *K*_B_ values were calculated by nonlinear regression analysis. Except for the Eurofins Cerep screens (see Radioligand Binding and Enzyme Inhibition methods), at least two independent assays (*N* = 2 biological replicates) generating a minimum of four technical replicates (the number of replicates for each data point included in the data analyses) were conducted for each experiment; specific replicate numbers are provided in figure legends. Biological replicates of only two were limited to confirmatory experiments. Data from cAMP assays were normalized to percent change of forskolin-stimulated cAMP production and fitted with a bell-shaped model. Data from the α_2A_R TRUPATH BRET2 dose–response tests were normalized to the percent maximal response of clonidine (a partial agonist, relative to epinephrine) using methods published by the developers.^81^ α_2B_R and α_2C_R TRUPATH BRET2 results comparing the effects of 10 μM clonidine and mitragynine to buffer (basal activity) were analyzed by one-way ANOVA with Dunnett’s multiple comparison test. In the tests of α_2A_Rs, we also conducted *t* tests between mitragynine and buffer (vehicle), where we observed effects that might indicate weak agonism. For ERK Western blots, blot density was quantified by ImageJ, and to account for differences in protein loading in each respective lane, the blot density was normalized to the log2 value of the pERK over the respective total ERK density. Data are expressed as fold-change over the buffer-treated condition. Statistical analysis was performed via *t*-test between treatment and buffer, and between agonist and antagonist plus agonist groups.

## Supplementary Material



## Notes

Additional in vitro pharmacological characterization of mitragynine and clonidine at α2-adrenoceptors, and binding and enzyme-inhibition profiling of mitragynine and its two major metabolites, 7-Hydroxymitragynine and 9-Hydroxycorynantheidine, at 99 targets (DOCX)

## #

Department of Pharmaceutical Sciences, Bouvé College of Health Sciences, Northeastern University, Boston, MA 02115
