# Supplementary material for: In Vitro Pharmacology of Mitragynine at α‑Adrenoceptors
Source: ACS Chem Neurosci. 2025 Nov 21;16(23):4531–45. doi: 10.1021/acschemneuro.5c00719 (PMC12679550; doi:10.1021/acschemneuro.5c00719)
Supplement: Supplementary file 1 [file cn5c00719_si_001.docx]

**Supporting Information for Publication**

In Vitro Pharmacology of Mitragynine at α-Adrenoceptors

Yiming Chen^1^, Jordan Seto^1^, Samuel Obeng^2^, Marco Mottinelli^3*^, Sushobhan Mukhopadhyay^3^, Richa Tyagi^1^, Aidan J. Hampson^4^, Christopher R. McCurdy^3^, Lance R. McMahon^2^, Nader H. Moniri^1,5^, Clinton E. Canal^1,5^

^1^Department of Pharmaceutical Sciences, College of Pharmacy, Mercer University, Atlanta, GA 30341

^2^Department of Pharmaceutical Sciences, Jerry H. Hodge School of Pharmacy, Texas Tech University Health Sciences Center, Amarillo, TX 79106

^3^Department of Medicinal Chemistry, College of Pharmacy, University of Florida, Gainesville, FL 32610

^4^Division of Therapeutics and Medical Consequences, National Institute on Drug Abuse, National Institutes of Health, Bethesda, Maryland 20892, USA

^5^Department of Biomedical Sciences, School of Medicine, Mercer University, Macon, GA 31207

*Current address: Department of Pharmaceutical Sciences, Bouvé College of Health Sciences, Northeastern University, Boston, MA 02115

**Figure S1.** Mitragynine and clonidine activity at α_2A_R–Gα_s(short)_ in HEK293 cells using TRUPATH BRET2 biosensors. Clonidine activated human α_2A_R–Gα_s_, albeit with ~6-fold lower efficacy than Gα_i1_; mitragynine, however, did not activate α_2A_R–Gα_s_. Data were normalized to the maximal response of clonidine at α_2A_R–Gα_i1_. Each symbol represents the mean ± SEM. At least three independent α_2A_R–Gα_s_ assays were conducted, generating 24 and 20 technical replicates of each concentration of clonidine and mitragynine, respectively. In this experiment, clonidine at α_2A_R–Gα_i1_ was tested once in duplicate for a direct comparison to α_2A_R–Gα_s_. Buf = buffer.

**Figure S2.** Mitragynine and clonidine activity at rat α_2A_R–cAMP in HEK293 cells. Clonidine attenuated forskolin-induced cAMP production in a concentration-dependent manner, whereas mitragynine showed no significant functional effects. Note that clonidine caused an increase in cAMP production at concentrations above 10 nM, consistent with observations of a Gα_i/o_ to Gα_s_ functional switch. Data show the percent of forskolin-induced cAMP accumulation. Symbols represent means ± SEMs. Two independent assays were conducted, generating 4 and 6 technical replicates of each concentration of clonidine and mitragynine, respectively. Buf = buffer.

**Figure S3.** Screening of 10 µM mitragynine and clonidine at α_2C_R–Gα_i1_ using TRUPATH BRET2 biosensors. HEK293 cells expressing human α_2C_R–Gα_i1_ were incubated with both drugs for 30 min before adding coelenterazine and measuring BRET. Mitragynine did not activate α_2C_R–Gα_i1_, regardless of this longer incubation time (see Figure 3). Note that the BRET signal window narrowed with longer intervals between adding coelenterazine and measuring BRET, suggesting substrate oxidation. Technical replicates are shown as individual data points in the violin plots. **** P < 0.0001. Buf = buffer; Clon = clonidine, MG = mitragynine.

**Figure S4.** In vitro binding and enzyme inhibition profile of 100 nM (1.0E-07) and 10 µM (1.0E-05) **7-Hydroxymitragynine**, an active mitragynine metabolite. NSI = Test compound interfered non-specifically in the assay.

**Figure S4 (continued).** In vitro binding profile of **7-Hydroxymitragynine**.

**Figure S4 (continued).** In vitro enzyme inhibition profile of **7-Hydroxymitragynine**.

**Figure S4 (continued).** Reference compound results.

**Figure S4 (continued).** Reference compound results.

**Figure S4 (continued).** Reference compound results.

**Figure S4 (continued).** Reference compound results.

**Figure S4 (continued).** Radioligands, target sources, and binding assay test conditions.

**Figure S4 (continued).** Radioligands, target sources, and binding assay test conditions.

**Figure S4 (continued).** Radioligands, target sources, and binding assay test conditions.

**Figure S4 (continued).** Radioligands, target sources, and binding assay test conditions.

**Figure S4 (continued).** Enzyme assay target sources and test conditions.

**Figure S4 (continued).** Radioligands, target sources, and binding assay test conditions.

**Figure S5.** In vitro binding and enzyme inhibition profile of 100 nM (1.0E-07) and 10 µM (1.0E-05) **9-Hydroxycorynantheidine**, an active metabolite of mitragynine. NSI = Test compound interfered non-specifically in the assay. Reference compounds and results, radioligands, target sources, binding and enzyme assay test conditions are the same as reported in Figure S4.

**Figure S5 (continued).** In vitro binding profile of **9-Hydroxycorynantheidine**.

**Figure S5 (continued).** In vitro enzyme inhibition profile of **9-Hydroxycorynantheidine**.

**Figure S6.** In vitro binding and enzyme inhibition profile of 100 nM (1.0E-07) and 10 µM (1.0E-05) **mitragynine**. NSI = Test compound interfered non-specifically in the assay. Reference compounds and results, radioligands, target sources, binding and enzyme assay test conditions are the same as reported in Figure S4.****

**Figure S6 (continued).** In vitro binding profile of **mitragynine**.

**Figure S5 (continued).** In vitro enzyme inhibition profile of **mitragynine**.
